# Efficacy and safety of dacomitinib in treatment-naïve patients with advanced NSCLC harboring uncommon EGFR mutation: an ambispective cohort study

**DOI:** 10.1186/s12885-023-11465-2

**Published:** 2023-10-16

**Authors:** Xingxiang Pu, Yu Zhou, Yi Kong, Bolin Chen, Aifang Yang, Jia Li, Kang Li, Yan Xu, Lin Wu

**Affiliations:** 1 The Second Department of Thoracic Oncology, Hunan Cancer Hospital, The Affiliated Cancer Hospital of Xiangya School of Medicine, Central South University, Changsha, 410000 People’s Republic of China; 2 The Department of Radiotherapy, Hunan Cancer Hospital, The Affiliated Cancer Hospital of Xiangya School of Medicine, Central South University, Changsha, 410000 People’s Republic of China

**Keywords:** Uncommon *EGFR* mutation, Non-small cell lung cancer, Dacomitinib, Efficacy, Safety

## Abstract

**Background:**

About 10% of non-small cell lung cancer (NSCLC) patients with epidermal growth factor receptor (*EGFR*) mutations are harbored as uncommon mutations. This study aimed to explore the efficacy and safety of dacomitinib, a second-generation *EGFR* tyrosine kinase inhibitor (EGFR-TKIs), in treating uncommon *EGFR*-mutated advanced NSCLC.

**Methods:**

Treatment-naïve advanced NSCLC patients treated with dacomitinib at Hunan Cancer Hospital with uncommon *EGFR* mutations were evaluated. The primary endpoint was progression-free survival (PFS). Secondary end points included overall survival (OS), objective response rate (ORR), disease control rate (DCR) and safety.

**Result:**

Between December 2019 and December 2021, a total of 16 patients was included. Median PFS was 14.0 (95% CI 4.32–23.7) months, and median OS was not reached. ORR was 68.8% (95% CI 41.3 to 89.0%) and DCR was 93.8% (95%CI 69.8 to 99.8%), including three achieving complete remission (CR) and eight achieving partial remission (PR). Median PFS for patients with brain metastasis was 9.0 (95%CI 6.9 to 11.1) months. Intracranial ORR was 100%, including 2 CR and 4 PR. Major treatment-related adverse events (TRAEs) included rash (87.5%), paronychia (62.5%), oral ulcers (50.0%), and diarrhea (50.0%), none of which were ≥ grade 3 TRAEs.

**Conclusions:**

Dacomitinib showed good activity and manageable toxicity in NSCLC patients with uncommon *EGFR* mutations.

**Supplementary Information:**

The online version contains supplementary material available at 10.1186/s12885-023-11465-2.

## Introduction

Lung cancer is the second most common cancer worldwide and the leading cause of cancer death [1]. Approximately 85% of all lung cancers are non-small cell lung cancer (NSCLC) [2].In advanced non-small cell lung cancer (NSCLC), epidermal growth factor receptor (EGFR) mutations are presented in approximately 40% of Asian populations and approximately 10% to 15% in non-Asian populations [3, 4]. *EGFR* exon 19 deletion and 21 L858R mutation are the most common *EGFR* alterations, which account for about 85% to 90% of *EGFR* mutations, considered as common *EGFR* mutations [5]. Meanwhile, approximately 10% of NSCLC patients with *EGFR* mutations harbored as uncommon mutations. Major uncommon *EGFR* mutation included G719X, S768I, and L861Q.

On the one hand, major large prospective clinical studies exploring the efficacy of *EGFR* tyrosine kinase inhibitors (EGFR-TKIs) in the first-line treatment of advanced NSCLC were restricted to common *EGFR* mutations, including FLAURA study (osimertinib versus gefitinib or erlotinib) [6], and ARCHER 1050 study (dacomitinib versus gefitinib) [7]. Common *EGFR* mutations showed efficacy to different generations of EGFR-TKIs, including the first- to third- generation EGFR-TKIs, while evidence of applying EGFR-TKIs in uncommon mutations were limited [3, 8, 9]. On the other hand, there were studies reported the activity of second-generation EGFR-TKI afatinib in treating uncommon *EGFR* mutations, which encourage the exploration of other second-generation EGFR-TKIs in treating advanced NSCLC harboring uncommon *EGFR* mutations [10].

Dacomitinib, a highly selective, irreversible second-generation EGFR-TKI, inhibits all human *EGFR* signaling. ARCHER 1050 study indicated that compared to gefitinib, dacomitinib showed a significant improvement in progression-free survival (PFS) in treating patients with *EGFR*-mutation-positive NSCLC (14.7 for dacomitinib vs. 9.2 months for gefitinib, hazard ratio [HR] 0.59; 95% confidence interval [CI] 0.47 to 0.74; *P* < 0.0001), leading to the approval of dacomitinib as the new standard first-line treatment for patients with *EGFR* exon 19 deletion and L858R mutation positive NSCLC by China National Medical Products Administration in 2019 [7]. Although some phase I/II clinical trial explored the application of dacomitinib in uncommon mutations [11, 12], there is limited evidence for dacomitinib treating advanced NSCLC patients with uncommon *EGFR* mutations in a real-world setting. Therefore, in this study, we conducted a real-world, ambispective cohort study exploring the efficacy and safety of dacomitinib in treating NSCLC patients with uncommon *EGFR* mutations.

## Materials and method

### Study design and eligibility criteria

Treatment-naïve patients with advanced NSCLC treated in the Department of Thoracic Oncology, Hunan Cancer Hospital between December 2019 and June 2022 were screened for *EGFR* mutation type. Eligible patients were pathological confirmed unresectable stage III or stage IV NSCLC with uncommon *EGFR* mutation (mutation other than exon 19 deletion and Leu858Arg point mutation in exon 21 [L858R]); with at least one measurable target lesion; receiving dacomitinib as first-line therapy; Eastern Cooperative Oncology Group (ECOG) Performance Status (PS) 0–2; and adequate organ and bone marrow function. Exclusion criteria were as follows: concomitant cancer or serious disease; previous exposure to any other EGFR-TKIs, radiation therapy or chemotherapy; follow-up data not available; or uncontrolled symptomatic brain metastasis.

The tests of *EGFR* mutations were conducted in genetic testing laboratory of Hunan Cancer Hospital and were identified using one of the following local test methods: peptide nucleic acid–mediated polymerase chain reaction clamping, direct sequencing, and/or next-generation sequencing.

This study protocol was confirmed by the Ethics Committee of Hunan Cancer Hospital and conducted in accordance with the Declaration of Helsinki. Each patient participating in the study signed informed consent form.

### Treatment plan

All patients were treated with dacomitinib. Initial doses of dacomitinib were 30 mg (for elderly patients or patients with inferior ECOG PS status assessed by clinicians) or 45 mg per day, administered orally once a day until disease progression or intolerant side effects developed. When grade 3–4 adverse events (AEs) occurred, treatment would be suspended until patients recovered to no more than grade 1 AE and the dose of dacomitinib should be adjusted to the lower level (45 mg adjusted to 30 mg and 30 mg adjusted to 15 mg) afterward. For patients with grade 2 AEs, dose adjustment was not necessary unless grade 2 AEs recurred.

### Response assessment and evaluation of adverse reactions

Computed tomography (CT) scans or magnetic resonance imaging (MRI) were applied to evaluate treatment response before and during dacomitinib treatment. Target lesions were assessed every two cycles (6 weeks). Imaging results were recorded and response was evaluated per modified Response Evaluation Criteria in Solid Tumors (mRECIST, version 1.1) [13]. Telephone follow-up was conducted every 3 months. Adverse events were recorded and assessed using Common Terminology Criteria for Adverse Events (CTCAE) version 4.03.

### Outcomes

The primary endpoint was progression-free survival (PFS), defined as the time interval from treatment to disease progression or death. Secondary endpoints included objective response rate (ORR, defined as the percentage of patients who achieved complete remission [CR] and partial remission [PR]), disease control rate (DCR, defined as the percentage of patients who achieved CR, PR, and stable disease [SD]), overall survival (OS, defined as the time interval from treatment to death of any cause) and safety.

### Statistical analysis

SPSS software (version 26, IBM) was used for all the statistical analysis. GraphPad Prism software (version 9, GraphPad Software, San Diego, CA, United States) was used for visualization. Categorical variables were presented as numbers and percentage and compared using the Chi-squared test and Fisher’s exact test. Continuous variables were presented as median and range and compared using Mann–Whitney U-test. Survival data were analyzed using the Kaplan–Meier method and compared using log-rank test. *P*-value < 0.05 indicates a statistically significant difference.

## Results

### Baseline characteristics

**Fig. 1:**
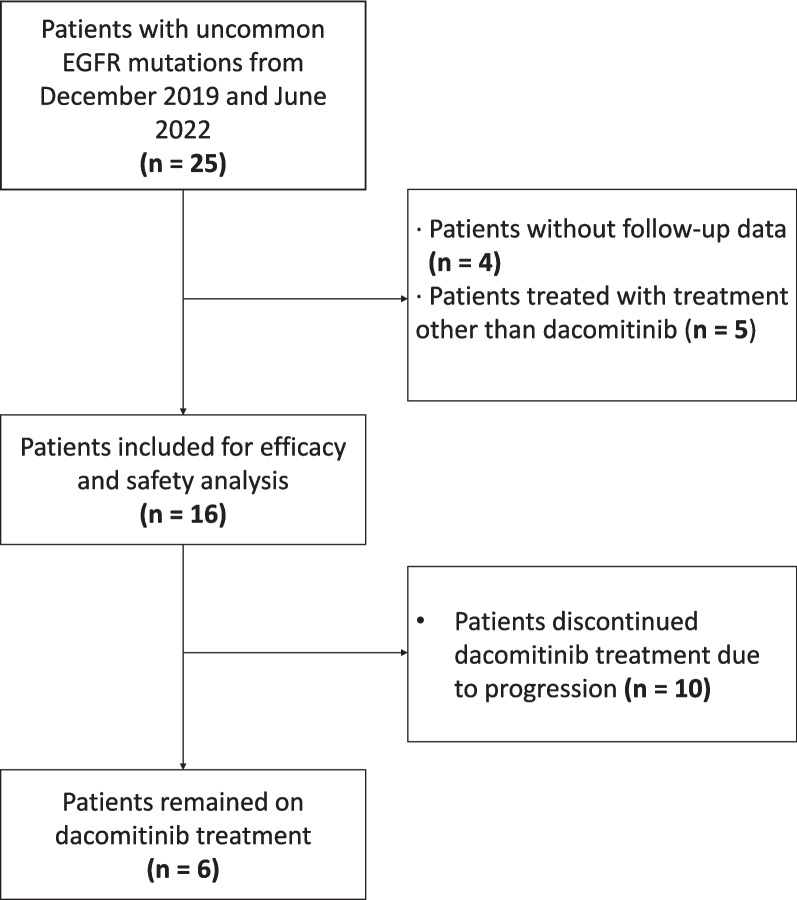
Flowchart of the enrolled schedule

Between December 2019 and June 2022, a total of 16 patients were included in this study for efficacy and safety retrospectively or prospectively, all of whom were treatment-naïve (Fig. 1). Baseline characteristics of the 16 patients were shown in Table 1. Among the 16 patients, the median age was 57 years (range 43–74 years), and including seven male patients and nine female patients. Overall, two (12.5%) patients were with unresectable stage III and 14 (87.5%) patients were with stage IV disease. A total of four (25.0%) patients were with ECOG PS 0 and 12 (75.0%) patients were with ECOG PS 1. Non-smokers or smokers/former smokers were found in 11 (68.8%) and 5 (31.3%) patients, respectively. The most common histology was adenocarcinoma (93.8%, 15/16). There were 8 (50.0%) patients with single-site metastasis, 6 (58.8%) patients with multi-site metastasis, and 2 (12.5%) patients without metastasis. To be noted, a total of 7 (43.8%) patients with brain metastases wasincluded in this study. All patients were detected for *EGFR* using next-generation sequencing in the central genetic testing laboratory. For the included *EGFR* mutation type, there were nine (56.3%) patients harboring 18 G719X mutation, of whom four (25.0%) with only 18 G719X mutation, three (18.8%) with 18 G719X and 18 E709X, as well as two (12.5%) with 18 G719X + 20 S768I. The rest uncommon mutation included one (6.3%) with 19 delins, two (12.5%) with 21 L833V/H835L and four (25.0%) with 21 L861Q. There were nine (56.3%) patients with accompanying mutation while the rest seven(43.8%) patients without. Four patients were enrolled retrospectively and the rest 12 patients were enrolled prospectively. The baseline characteristics of patients enrolled prospectively and retrospectively were balanced. Most patients (12/16, 75.0%) received dacomitinib 45 mg per day as initial treatment.

**Table 1:** Characteristics of enrolled patients and treatment (*N* = 16)

Characteristics	Total, n (%)	Retrospectively enrolled, n (%)	Prospectively enrolled, n (%)	*P* value
Median age, years (range)	57 (43–74)	65 (52–74)	55 (43–68)	0.05
Gender				0.192
Male	7 (43.8)	3 (18.8)	4 (25.0)	
Female	9 (56.3)	1 (6.3)	8 (50.0)	
Pathological stage				0.450
III	2 (12.5)	1 (6.3)	1 (6.3)	
IV	14 (87.5)	3 (18.8)	11 (68.8)	
ECOG PS score				0.755
0	4 (25.0)	1(6.3)	3 (18.8)	
1	12 (75.0)	3 (18.8)	9 (56.3)	
Smoking status				0.365
Nonsmoker	11 (68.8)	2 (12.5)	9 (56.3)	
Smoker/former smoker	5 (31.3)	2 (12.5)	3 (18.8)	
Histology				0.750
Adenocarcinoma	15 (93.8)	4 (25.0)	11 (68.8)	
Adenosquamous carcinoma	1 (6.3)	0 (0)	1 (6.3)	
Metastatic sites				0.641
Single site metastasis	8 (50.0)	2 (12.5)	6 (37.5)	
Multiple site metastasis	6 (37.5)	1 (6.3)	5 (31.3)	
No distant metastasis	2 (12.5)	1 (6.3)	1 (6.3)	
Brain metastasis				0.608
Yes	7 (43.8)	2 (12.5)	5 (31.3)	
No	9 (56.3)	2 (12.5)	7 (43.8)	
*EGFR* mutation				0.859
18 G719X	4 (25.0)	0 (0)	4(25.0)	
18 G719X + 18 E709X	3 (18.8)	1 (6.3)	2 (12.5)	
18 G719X + 20 S768I	2 (12.5)	1 (6.3)	1 (6.3)	
19 delins	1 (6.3)	0 (0)	1 (6.3)	
21 L833V/H835L	2 (12.5)	1 (6.3)	1 (6.3)	
21 L861Q	4 (25.0)	1 (6.3)	3 (18.8)	
PD-L1 expression				0.550
≥ 1%	2 (12.5)	0 (0)	2 (12.5)	
< 1%	14 (87.5)	4 (25.0)	10 (62.5)	
Accompanying mutation				0.392
Yes	9 (56.3)	3 (19.8)	6 (37.5)	
No	7 (43.8)	1 (6.3)	6 (37.5)	
Initial dosage of dacomitinib				0.755
30 mg	4 (25.0)	1(6.3)	3 (18.8)	
45 mg	12 (75.0)	3 (18.8)	9 (56.3)	
Dose Reduction				0.511
Yes	6 (37.5)	1 (6.3)	5 (31.3)	
No	10 (62.5)	3 (18.8)	7 (43.8)	

Data are presented as number (%) unless otherwise indicated

*Abbreviations:* *ECOG PS* Eastern Cooperative Oncology Group performance status, *EGFR* Epidermal growth factor receptor, *PD-L1* Programmed cell death-ligand 1

### Efficacy

**Fig. 2:**
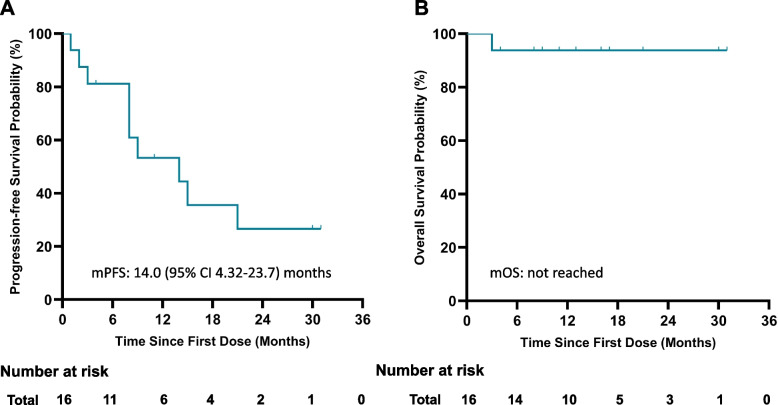
Kaplan Meier Curve for progression-free survival and overall survival of whole group

Data cutoff date of this study was February 13th, 2023. The median follow-up time for all patients was 16.5 (range: 3.6 to 32.1) months. Median PFS of the whole group was 14.0 (95% CI 4.32–23.7) months, and the median OS was not reached (Fig. 2A and B). Subgroup analysis was further performed. For different major *EGFR* mutation types, the median PFS varied. The median PFS for 18 G719X subgroup was 14.0 (95%CI 4.2 to 23.8) months while the median PFS for 21 L861Q subgroup was 6.5 (95%CI 0 to 20.7) months. Seven patients with brain metastasis were enrolled and six with evaluable intracranial lesion. The median PFS for these patients was relatively short, with median PFS 9.0 (95%CI 6.9 to 11.1) months (Table 2). Additionally, for patients enrolled prospectively or retrospectively, there was no significant difference in survival outcomes (Supplement Figure S1A and B). For patients started with different initial dose, there was no significant difference found in PFS, as so for patients with or without experiencing dose reduction (Supplement Figure S1C and D).

**Table 2:** Treatment responses of whole group and different subgroups

	Objective response		Disease control		Median PFS
	No. (%)	95%CI	No. (%)	95%CI	Months (95% CI)
Whole group (*n* = 16)	11 (68.8)	41.3 to 89.0	15 (93.8)	69.8 to 99.8	14.0 (4.3 to 23.7)
Major *EGFR* mutation					
G719X (*n* = 9)	6 (66.7)	29.9 to 92.5	8 (88.9)	51.8 to 99.7	14.0 (4.2 to 23.8)
L861Q (*n* = 4)	2 (50.0)	6.8 to 93.2	4 (100.0)	39.8 to 100.0	6.5 (0 to 20.7)
Evaluable Brain metastasis (*n* = 6^a^)	5 (83.3)	35.9 to 99.6	6 (100.0)	54.1 to 100.0	9.0 (6.9 to 11.1)

*Abbreviations:* *CI *Confidence interval, *PFS *Progression-free survival, *EGFR *Epidermal growth factor receptor

^a^The ORR and DCR for these patients were intracranial ORR and DCR

**Fig. 3:**
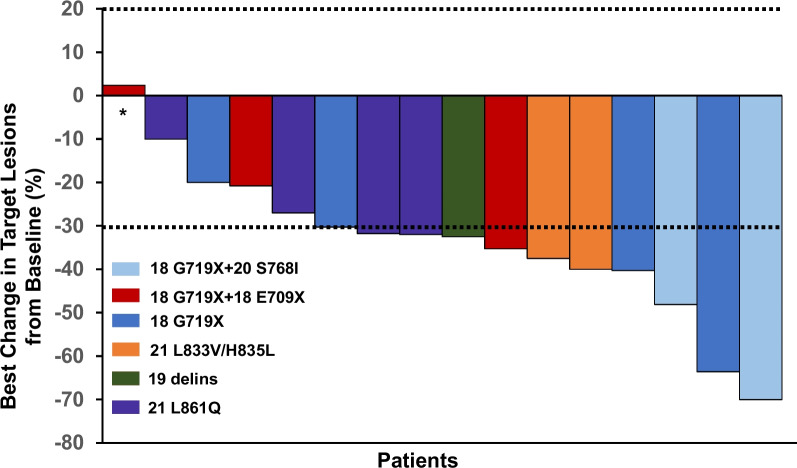
Best percentage change in target lesion size from baseline. Note: The upper dashed line at + 20% represents the threshold for progressive disease. The lower dashed line at -30% represents the boundary for partial response. * New lesion appeared in this patient

**Fig. 4:**
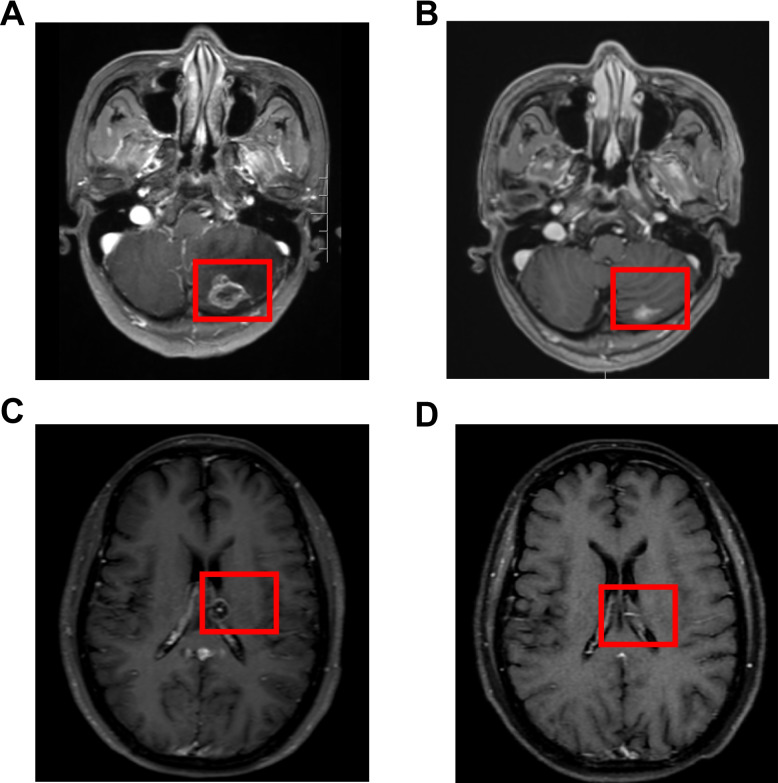
The best change of intracranial lesions in two patients with baseline intracranial lesions over 1 cm (**A**) baseline intracranial lesion of patient 1; **B** best change of intracranial lesion of patient 1; **C** baseline intracranial lesion of patient 2; **D** best change of intracranial lesion of patient 2

ORR of the whole group was 68.8% (95%CI 41.3 to 89.0%), including three (18.8%) patient experienced CR and eight (50.0%) patients experienced PR. The rest four (25.0%) patients achieved SD and one (6.2%) patient experienced disease progression. DCR of the whole cohort was 93.8% (95%CI 69.8 to 99.8%). For the major EGFR mutation types, ORR of patients with G719X was 66.7% (95%CI 29.9 to 92.5%) and DCR was 88.9% (95%CI 51.8 to 99.7%). For the four patients with L861Q mutation, ORR was 50.0% (95%CI 6.8 to 93.2%) and DCR was 100% (95%CI 39.8 to 100.0%). The waterfall plot of the percent change in optimal target lesions in the lungs for 16 patients along with the corresponding *EGFR* mutation type was shown in Fig. 3. For the six patients with evaluable brain metastasis, intracranial ORR was 85.7% (95%CI 35.9 to 99.6%) and intracranial DCR was 100% (95%CI 54.1 to 100%), including one patient achieving CR and four patients with PR. Of note, there two patients with the maximum diameter of baseline intracranial lesions over 1 cm. These two patients were both harboring with G719X mutation. These two patients both achieved PR for intracranial lesions, and the intracranial time to response was 85 days and 27 days, respectively. The best change of the intracranial lesions of these two patients were shown in Fig. 4.

### Subsequent treatment

At the data cut-off date, 10 of 16 patients experienced systematic disease progression. Of these 10 patients, eight patients were with local progression and two patients were with distributed progression. Six patients experienced newly diagnosed intracranial disease. Of these ten patients with disease progression, four patients took third-generation EGFR-TKI as subsequent treatment. Three patients received immunotherapy combined with platinum-based chemotherapy with bevacizumab. Two patients with only brain metastasis received local radiotherapy. One patient refused the above treatment and took Chinese traditional herbs as subsequent treatment.

### Safety profile

Treatment-related adverse events (TRAEs) emerged during dacomitinib treatment were observed in 15 of the whole 16 patients (93.8%). Overall, all the TRAEs were grade 1 and grade 2. None of them experience ≥ grade 3 TRAE. as shown in Table 3, common TRAEs were rash (87.5%), paronychia (62.5%), oral ulcers (50.0%), and diarrhea (50.0%). Specifically, none of these patients experienced interstitial pneumonia (0%). During the treatment, six (37.5%) patients experienced dose reduction due to intolerance of grade 2 AEs, including one patient with grade 2 oral ulcer, two patients with grade 2 rash, one patient with grade 2 oral ulcer and rash, one patient with grade 2 oral ulcer, rash and paronychia. No patient discontinued dacomitinib treatment due to toxicities.

**Table 3:** Treatment-emergent adverse events (*N* = 16)

Adverse events	All Grade (n, %)	Grade 1 (n, %)	Grade 2 (n, %)	≥ Grade 3 (n, %)
Oral ulcer	8 (50.0)	4 (25.0)	4 (25.0)	0
Rash	14 (87.5)	6 (37.5)	8 (50.0)	0
Paronychia	10 (62.5)	9 (56.3)	1 (6.2)	0
Diarrhea	8 (50.0)	7 (43.8)	1 (6.3)	0
Interstitial pneumonia	0	0	0	0

## Discussion

In this study, we reported the efficacy and safety results of 16 patients with advanced NSCLC harboring major uncommon EGFR mutations receiving the second-generation EGFR-TKI dacomitinib as first-line therapy. Overall, the ORR was 68.8%, DCR was 93.8% and median PFS was 14.0 months. The high response rate and survival results indicated that dacomitinib could be one of the treatment choices for patients with uncommon *EGFR* mutation.

EGFR-TKIs are recognized as standard first-line treatment option for the treatment of patients with common EGFR-mutated advanced NSCLC. Common EGFR mutations, including *EGFR* exon 19 deletion and L858R,were considered as sensitive to EGFR-TKIs, while uncommon *EGFR* mutations are considered less sensitive with low response and survival rates [14–19]. For first-generation EGFR-TKIs, including gefitinib and erlotinib, previous studies reported in treating patients with major uncommon an ORR of 25.7% to 48.8%, with median PFS of 5.0 to 6.0 months [14, 20, 21]. For major uncommon *EGFR* mutations, including L861Q, G719X, and S768I, a study reported that gefitinib or erlotinib generated an ORRs of 33.3 to 43.1% and median PFS of 2.2 to 7.7 months [17]. On the other hand, compared to first-generation of EGFR-TKI, clinical studies and real-world data showed that second- and third- generation of EGFR-TKIs might be more effective for NSCLC patients with uncommon *EGFR* mutations. Afatinib was approved as first-line treatment for patients with *EGFR* L861Q, G719X, and S768I mutation in 2018 by Food and Drug Administration of the United States. Wu et al. reported that the median PFS was significantly longer for patients harboring major uncommon *EGFR* mutations treated with afatinib compared to patients treated with gefitinib and erlotinib (median PFS for gefitinib group versus erlotinib group versus afatinib group: 3.0 months versus 0.9 months versus 10.5 months; *p* = 0.013) [8]. Additionally, the combined post-analysis of the three LUX-lung trials showed that for patients harboring major uncommon mutations treated with afatinib, including G719X, L861Q, and S768I, the ORRs were 77.8%, 56.3%, and 100%, respectively, with median PFS of 13.8 months, 8.2 months and 14.7 months, respectively [22]. The differences of efficacy between first and second generations of EGFR-TKIs mainly contributed to different molecular mechanisms. The first-generation EGFR-TKIs block *EGFR* activity in an ATP-competitive and -reversible manner, while second-generation EGFR-TKIs could generate covalent binding to *EGFR* at Cys797 residue, which led to the irreversible inhibition of the *EGFR* [23]. Moreover, second-generation EGFR-TKIs could irreversible inhibit human epidermal growth factor receptor (*HER*) 2 and *HER*3, which could explain the better efficacy of afatinib in treating patients harboring uncommon *EGFR* mutations compared to first-generation EGFR-TKIs.

Similar to afatinib, dacomitinib is an inversible, second-generation TKI. In a single-arm, ambispective study in China, a total of 32 patients with NSCLC harboring major uncommon *EGFR* mutations treated with dacomitinib, among whom 18 receiving dacomitinib as first-line treatment [24]. Results showed that in first-line settings, patients had ORR of 72.2% (13/18) and DCR of 100%(18/18).Median PFS not reached in this study. For patients with major uncommon mutations showed different ORR (G719X versus L861X versus S768I: 56.5% vs 44.4% vs 62.5%) and median PFS (10.3 months for G719X versus not reached for L861X versus 6.5 months for S768I). Results of this study of dacomitinib were comparable with the results of our real-world study, in which the ORR of whole group was 68.8%, median PFS 14.0 months, ORR of patients with G719X was 66.7% and 50.0% of patients with L861Q mutation.

Furthermore, third-generation EGFR-TKI has also shown the efficacy in advanced NSCLC patients with uncommon mutations. In the KCSG-LU15-09 trial by Cho et al., among 32 patients with uncommon *EGFR* mutations G719X, L861Q and S768I, the ORRs of osimertinib was 53%, 78%, 38%, and median PFS of 8.2 months, 15.2 months, and 12.3 months, respectively [25]. UNICORN study, a multicenter, retrospective study, further explored the efficacy of osimertinib in treating uncommon EGFR mutations (exon 20 insertions excluded) [26]. A total of 60 patients were included. For patients with G719X, ORR was 47%, mPFS 8.8 months, and mDoR 9.1 months. For patients with L861Q, ORR was 80%, mPFS 16 months, and mDoR 16 months. There were some differences in efficacy between results of osimertinib, afatinib and dacomitinib. However, so far, most data of osimertinib were from real-world studies and revealed variable activity. There was no randomized controlled trial directly comparing the efficacy of osimertinib and the second-generation EGFR-TKIs in NSCLC patients harboring uncommon *EGFR* mutations. Prospective data is lacking and more clinical evidence is warranted.

Brain metastasis occurred in about 40–50% of advanced NSCLC during the disease course [27]. However, limited data have been reported on the efficacy of EGFR-TKIs for NSCLC patients harboring uncommon *EGFR*-mutant with brain metastases. Osimertinib has potential central nervous system activity for treatment response in NSCLC patients. A case report reported that one NSCLC patient with leptomeningeal metastases harboring uncommon *EGFR* mutations G719S and L861Q taking double-dose osimertinib treatment and achieved over 1-year stable disease [28]. Ma et al. reported that afatinib and osimertinib were effective in four of seven patients with uncommon EGFR mutations found in cerebrospinal fluid ctDNA [29]. KCSG-LU15-09 study reported that patients with uncommon EGFR mutations taking osimertinib 80 mg orally once per day could achieve an intracranial ORR of 40.0% (2/5) [25].Several studies have demonstrated the potential efficacy of dacomitinib in *EGFR*-positive NSCLC with CNS metastases, with ORR ranging from 87.5% to 92.9% and DCR of 100% [30–33]. A recent study by Li et al. showed that intracranial disease control was observed in 92.9% of advanced NSCLC patients with brain metastasis treated with dacomitinib (13/14) [34]. In our study, all six (100%) patients with evaluable brain metastasis demonstrated disease control of brain metastases. Results of our study further supported the efficacy of dacomitinib in NSCLC patients with brain metastases harboring uncommon mutations, with intracranial ORR of 85.7% and intracranial DCR of 100%.

Apart from efficacy, adverse events were another major concern in EGFR-TKI treatment. Compared with first-generation EGFR-TKIs, dacomitinib was associated with increased toxicity of diarrhea, rash, stomatitis, and paronychia. In this study, the adverse effects of dacomitinib were manageable, and there was no patient who discontinued treatment due to side effects. Overall adverse effects of dacomitinib in the treatment of uncommon EGFR mutations were within acceptable and tolerable ranges.

This study has some limitations. On the one hand, this study is a real-world ambispective analysis, clinical activity of dacomitinib remains to be explored in larger sample size, head-to head clinical study. In addition, as compound EGFR mutation showed different clinical profile [3, 35], efficacy of dacomitinib in treating patients harboring compound EGFR mutation should be further explored as there were relatively small number of these patients enrolled in this study. Also, selection bias is inevitable compared with prospective clinical trials.

## Conclusion

This study demonstrates the potential efficacy of dacomitinib in patients with advanced NSCLC harboring uncommon EGFR mutations. Moreover, dacomitinib has good efficacy in patients with brain metastases and a relatively low risk of adverse effects. Dacomitinib may be a new treatment option for patients harboring uncommon EGFR mutations in first-line settings.

### Supplementary Information

**Additional file 1.**

## Data Availability

The original data can be acquired from the corresponding authors under reasonable requirement.
