# Supplementary material for: Efficacy and safety of dacomitinib in treatment-naïve patients with advanced NSCLC harboring uncommon EGFR mutation: an ambispective cohort study
Source: BMC Cancer. 2023 Oct 16;23:982. doi: 10.1186/s12885-023-11465-2 (PMC10577935; doi:10.1186/s12885-023-11465-2)

Supplementary Material

**Efficacy and safety of dacomitinib in treatment-naïve patients with advanced NSCLC harboring uncommon EGFR mutation: an ambispective cohort study**

Content

Figure S1. (A) Progression-free survival and (B) overall survival of patients enrolled retrospectively or prospectively; progression-free survival of (C) patients started with different initial dose and (D) with or without experiencing dose reduction. **2**

Figure S1. (A) Progression-free survival and (B) overall survival of patients enrolled retrospectively or prospectively; progression-free survival of (C) patients started with different initial dose and (D) with or without experiencing dose reduction.


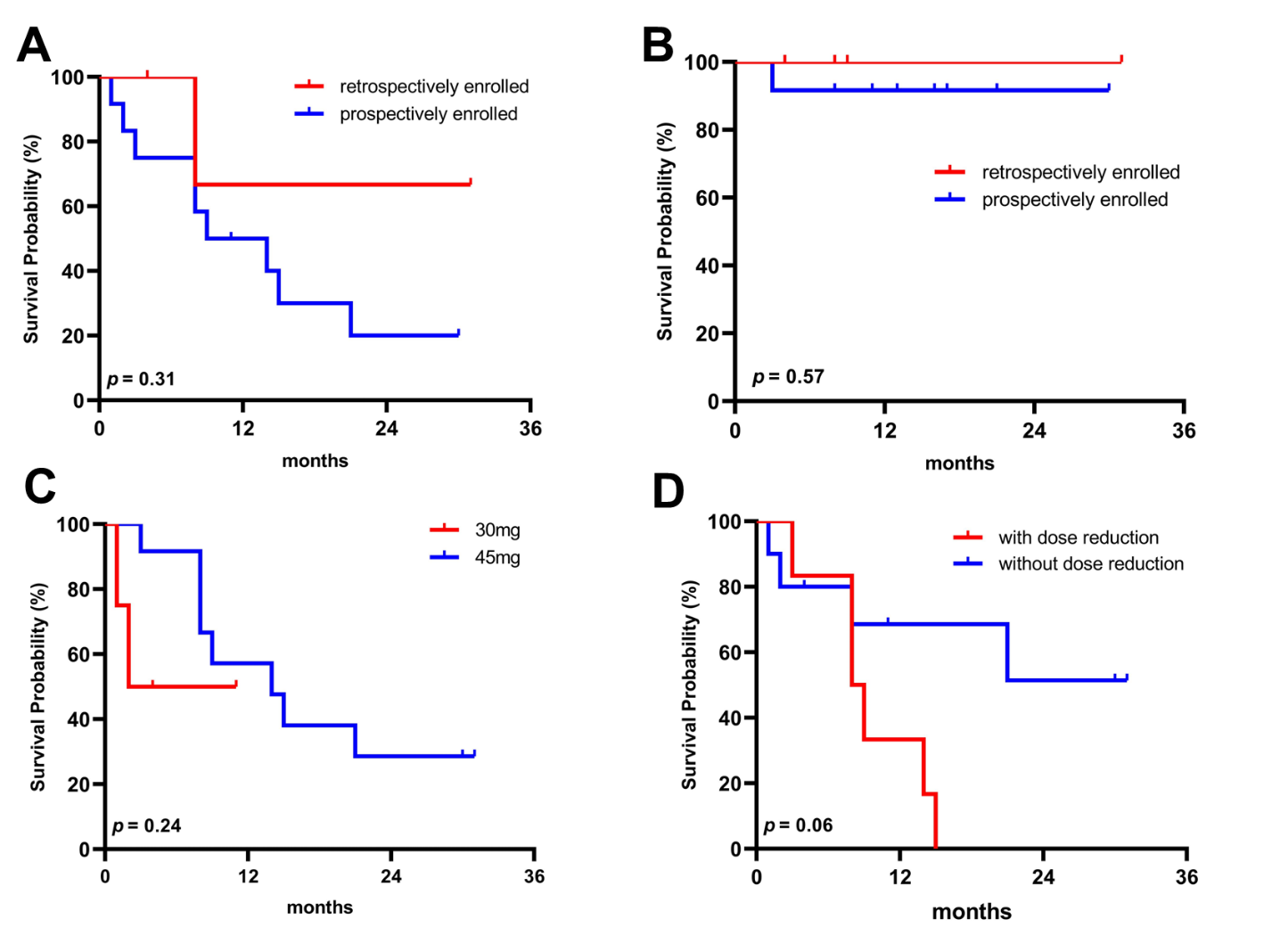

Supplement: Supplementary file 1 — Additional file 1. [file 12885_2023_11465_MOESM1_ESM.docx]
